# Identification of a Novel Epithelial–Mesenchymal Transition-Related Gene Signature for Endometrial Carcinoma Prognosis

**DOI:** 10.3390/genes13020216

**Published:** 2022-01-25

**Authors:** Tianyuan Ruan, Jing Wan, Qian Song, Peigen Chen, Xiaomao Li

**Affiliations:** Department of Gynecology, The Third Affiliated Hospital, Sun Yat-Sen University, Guangzhou 510630, China; rtiany@mail2.sysu.edu.cn (T.R.); wanjing@mail.sysu.edu.cn (J.W.); songq25@mail.sysu.edu.cn (Q.S.); chenpg@mail2.sysu.edu.cn (P.C.)

**Keywords:** endometrial cancer, epithelial–mesenchymal transition, immune microenvironment, immune checkpoint blockades, prognostic signature

## Abstract

(1) Background: Endometrial cancer is the most prevalent cause of gynecological malignant tumor worldwide. The prognosis of endometrial carcinoma patients with distant metastasis is poor. (2) Method: The RNA-Seq expression profile and corresponding clinical data were downloaded from the Cancer Genome Atlas database and the Gene Expression Omnibus databases. To predict patients’ overall survival, a 9 EMT-related genes prognosis risk model was built by machine learning algorithm and multivariate Cox regression. Expressions of nine genes were verified by RT-qPCR. Responses to immune checkpoint blockades therapy and drug sensitivity were separately evaluated in different group of patients with the risk model. (3) Endometrial carcinoma patients were assigned to the high- and low-risk groups according to the signature, and poorer overall survival and disease-free survival were showed in the high-risk group. This EMT-related gene signature was also significantly correlated with tumor purity and immune cell infiltration. In addition, eight chemical compounds, which may benefit the high-risk group, were screened out. (4) Conclusions: We identified a novel EMT-related gene signature for predicting the prognosis of EC patients. Our findings provide potential therapeutic targets and compounds for personalized treatment. This may facilitate decision making during endometrial carcinoma treatment.

## 1. Introduction

Endometrial carcinoma (EC) is one of the most prevalent causes of gynecological malignant tumors. The incidence of EC kept increasing 1.3% per year from 2007 to 2016 worldwide. According to the latest cancer statistics, it was estimated that there would be approximately 65,620 new cases diagnosed and 12,590 deaths in the United States in 2020 [1]. Those patients who are diagnosed in the early stage could receive appropriate treatment and achieve a good prognosis. However, EC patients with distant metastasis had poor prognosis, with a 5-year survival rate lower than 20% [2]. It is essential to implore efficient biomarkers and therapeutic targets to acquire better prognoses for EC patients.

Epithelial–mesenchymal transition (EMT) is a multistep process in which epithelial cells develop a mesenchymal phenotype characterized by the loss of polarity and barrier integrity, and therefore gain motility and invasive properties [3]. EMT plays a crucial role in tumor invasiveness and metastasis [4]. Accumulated evidence shows that EMT plays important role in cancer metastasis and drug resistance in multiple cancers, including endometrial cancer. A decreased level of E-cadherin was reported in endometrial cancer with an elevation of Snail and Slug nuclear expression, which significantly represented the EMT process and related to poor prognosis in endometrial cancer [5]. However, the expression profile of EMT-related genes as a prognostic factor and potential as a therapeutic target has not been systematically explored.

The EMT signaling pathways could be activated by several cytokines or growth factors from the focal microenvironment. The tumor cells that undergo EMT are also associated with immune exclusion and immune deviation in the tumor microenvironment (TME) [6]. Recently, pan-cancer analysis has revealed a strong correlation between EMT and immune activation [7]. The crosstalk between EMT and the immune cells may bring potential therapeutic opportunities.

In our study, we investigate the correlation between EMT-related genes (ERGs) expression and clinical data in 543 endometrial cancer patients downloaded from the Cancer Genome Atlas (TCGA). The least absolute shrinkage and selection operator (LASSO) Cox regression was carried out to constructed the EMT-related genes signature. We also conducted an integrated analysis of tumor immune microenvironment, immunotherapy response, and drug sensitivity according to the gene signature.

## 2. Method

### 2.1. Data Preparation

We acquired the EMT-related genes (ERGs) list from the epithelial–mesenchymal transition gene database (http://dbemt.bioinfo-minzhao.org/dbemt2.txt (accessed on 29 October 2020)), which contained 1184 epithelial–mesenchymal transition genes with experimentally verified information and pre-calculated regulation information for cancer metastasis [8]. Level 3 high-throughput sequencing mRNA expression data of ERGs and corresponding clinical data of the endometrial carcinoma (UCEC) cohort were obtained from the TCGA Data Portal (https://tcga-data.nci.nih.gov/tcga/ (accessed on 3 November 2020)). Somatic mutation data and copy number variation were also downloaded from TCGA. The fragments per kilobase of transcript per million method was applied to normalize the expression data. The gene expression dataset, GSE21882 based on GPL10422 SWEGENE H_v3.0.1 and GPL10423 SWEGENE H_v2.1.1 was downloaded from GEO (https://www.ncbi.nlm.nih.gov/geo/query/acc.cgi?acc=GSE21882 (accessed on 5 December 2020)). There were 45 type 1 endometrial carcinoma samples included in the dataset. The raw data were downloaded from the GEO database as a dataset soft file.

### 2.2. Differentially Expressed ERGs

The “limma” package [9] was applied to identify the differentially expressed EMT-related genes (EGRs). ERGs in the TCGA-UCEC mRNA expression cohort were identified with FDR < 0.05 and |logFC| > 1. Additionally, “ggplot”, “ggrepel”, and “heatmaps” [10,11,12] were executed in the R package to perform visualization.

### 2.3. Enrichment Analysis of Intersection Genes

GO (Gene Ontology) and KEGG (Kyoto Encyclopedia of Genes and Genomes) enrichment analyses and visualization of intersection genes were performed by “clusterProfiler”, “topGo”, “Rgraphviz” and “pathview” R package [13,14,15,16] with *p* < 0.05 as the cut-off value.

### 2.4. Individualized Gene Signature

As we acquired the ERGs and the clinical data of 539 patients in TCGA-UCEC, univariate COX regression was performed with “survival” R packages, and 101 prognostic-related ERGs in TCGA-UCEC were identified with *p* < 0.05. Next, we performed a machine learning algorithm, the LASSO algorithm [17] with a 10,000-time cross-validation. At last, 9 genes were identified as prognostic-related ERGs. With these 9 prognostic-related ERGs, multivariate Cox regression analysis was performed to generate an individual predictive formula. The risk score, (RS) = $\;\sum_{i=1}^{n}\beta_{i}*x_{i}\;$(β stands for the regression coefficient and *x* stands for the expression of a selected gene), based on the formula mentioned above were calculated in the TCGA-UCEC cohort and verified in the GSE21882 dataset. Based on the median value of RS, the patients in the two datasets were divided into low-risk and high-risk groups; then, overall survival, disease-free survival, and clinical–pathological characteristics were compared between the two groups.

### 2.5. Nomogram Construction

Univariate analysis has identified that age at diagnosis, FIGO stage, differentiation grade, and risk score were significantly associated with overall survival. The cut-off *p* was set as 0.05. All the significant prognostic variates mentioned above were enrolled in the multivariate Cox analysis. The *p* was set as 0.1 in the multivariate Cox analysis, then age at diagnosis, differentiation grade, FIGO stage, and the risk score were included in the nomogram construction. C-index was applied to evaluate the predictive power of the constructed nomogram for the overall survival [18]. A larger C-index dictated a better predictive power of the nomogram. The calibration curve was applied to determine the compliance between the predictive survival probability and the actual survival proportion after bias correction for the 3-year and 5-year overall survival.

### 2.6. Tumor Immune Microenvironment Analysis

ESTIMATE (estimation of stromal and immune cells in malignant tumor tissues using expression data) is one of the algorithms developed to evaluate the cell tumor composition by calculating the immune and stromal scores using Pearson’s correlation coefficient. By using the “estimate” R package [19], the immune and stromal scores were calculated based on the gene expression data of EC patients in TCGA-UCEC. The infiltration levels of 22 immune cells in each TCGA-UCEC patient were calculated through the R package “CIBERSORT” algorithm based on the gene expression levels [20].

### 2.7. Immunotherapy and Chemotherapy Response Prediction

CTLA-4 and PD-1/PD-L1 pathways have been proven to be crucial in immune evasion, and thereby immune checkpoint blockades therapy targeting PD-1 or CTLA-4 were developed to improve certain patients’ prognosis [21]. The tumor immune dysfunction and exclusion (TIDE) algorithm was utilized to predict the clinical response to immune checkpoint blockades therapy [22]. As chemotherapy is necessary for advanced EC patients and substantial chemotherapy was still debatable after the failure of initial chemotherapy, we acquired information from the Genomics of Drug Sensitivity in Cancer (GDSC) database [23]. Clinical response to the different compounds was predicted through the R package “pRRophetic” [17] and the half-maximal inhibitory concentration (IC50) of the samples were predicted by ridge regression [17].

### 2.8. PCR Validation

Quantitative real-time PCR was performed to validate the mRNA expression levels of the 9 ERGs. In total, 10 pairs of samples of EC patient’s tumor tissue and adjacent normal tissue were acquired from the third affiliated hospital of Sun Yat-sen University. TRIzol Reagent (Invitrogen, Carlsbad, CA, USA) was applied to extract the total RNA. PrimeScript^TM^ RT Reagent kit (Takara Biotech, Dalian, China) was applied to make cDNA according to the manufacturer instruction. Talent qPCR PreMix (SYBR-Green) (Tiangen Biotech, Beijing, China) was used to perform real-time quantification. A melting curve analysis was then performed. The expression levels were normalized by GAPDH. Relative expression (defined as the fold change) of the target genes were determined by the following equation: 2^−ΔΔCt^ (ΔCt = ΔCt^target^ − ΔCt^GAPDH^; ΔΔCt = ΔCt^tumor^ − ΔCt^nontumor^). This value was normalized to the average fold change in the normal endometrial tissues, which was defined as 1.0. The sequence of the primers was listed in Table 1.

## 3. Result

### 3.1. Identification of Differentially Expressed ERGs

**Figure 1:**
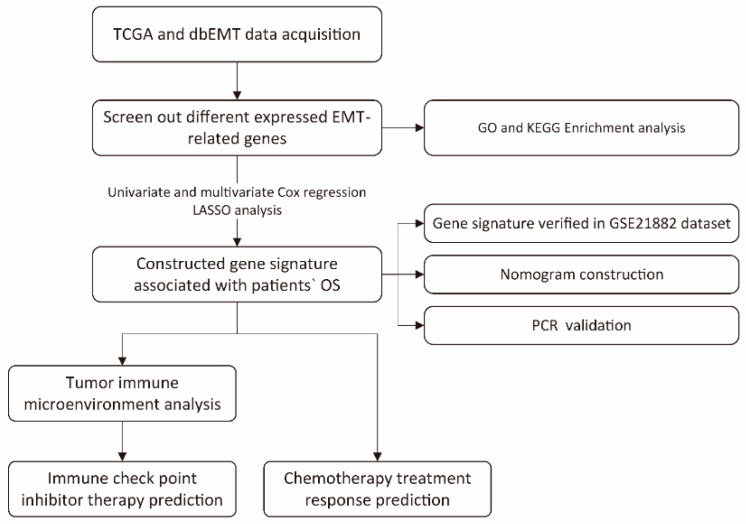
Study flowchart.

**Figure 2:**
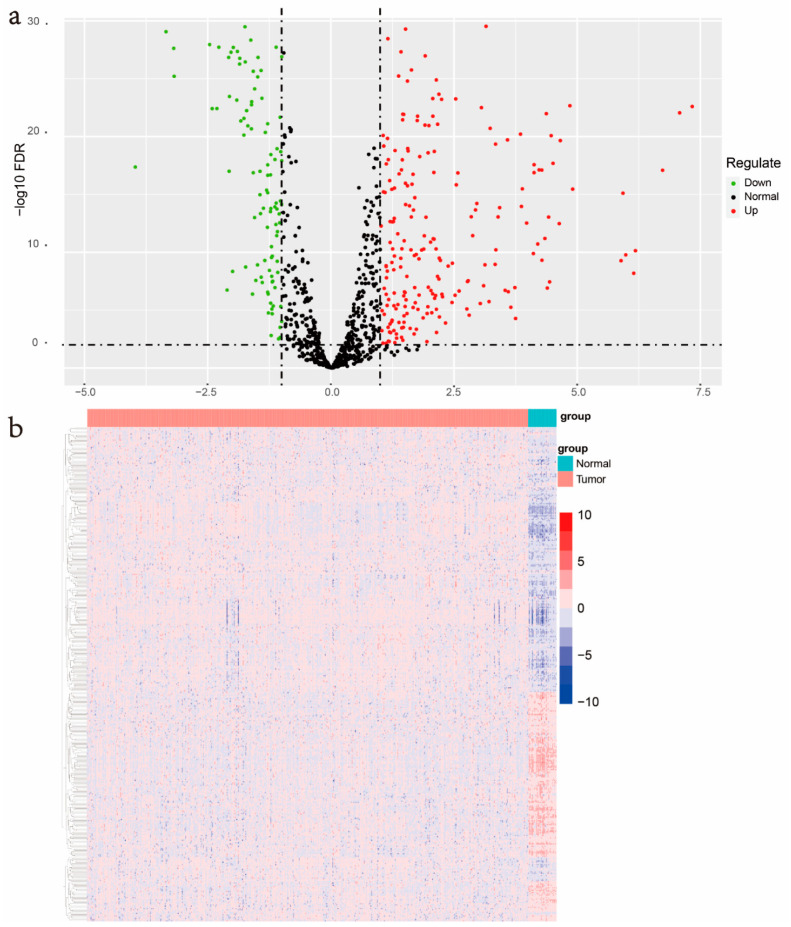
Identification of differently expressed EMT-related genes. (**a**) The volcano plot was generated for depicting the 1184 differently expressed EMT-related genes (ERGs) levels between uterine corpus endometrial carcinoma (UCEC) and normal endometrium tissues, with an FDR < 0.05, and |logFC| > 1. (**b**) Heatmap for all the screened ERGs expression in TCGA-UCEC dataset.

The overall process for constructing and verifying EMT-related gene signature is shown in Figure 1. RNA-seq and clinical follow-up data from TCGA-UCEC datasets were downloaded, which contains 543 cancer samples and 35 normal samples. A total 1184 EMT-related genes (ERGs) were acquired from the Human EMT Database. Then, the transcriptional expression of these 1184 ERGs in TCGA-UCEC RNA-seq data were compared between normal samples and UCEC samples through the “limma” package in R software (|logFC| > 1, FDR < 0.05). The results indicated that there were 236 genes significantly upregulated and 169 genes significantly downregulated in UCEC (Figure 2a). The screened differently expressed EMT-related genes were enrolled for heatmap generation (Figure 2b).

### 3.2. Biological Functions Pathways Analysis

**Figure 3:**
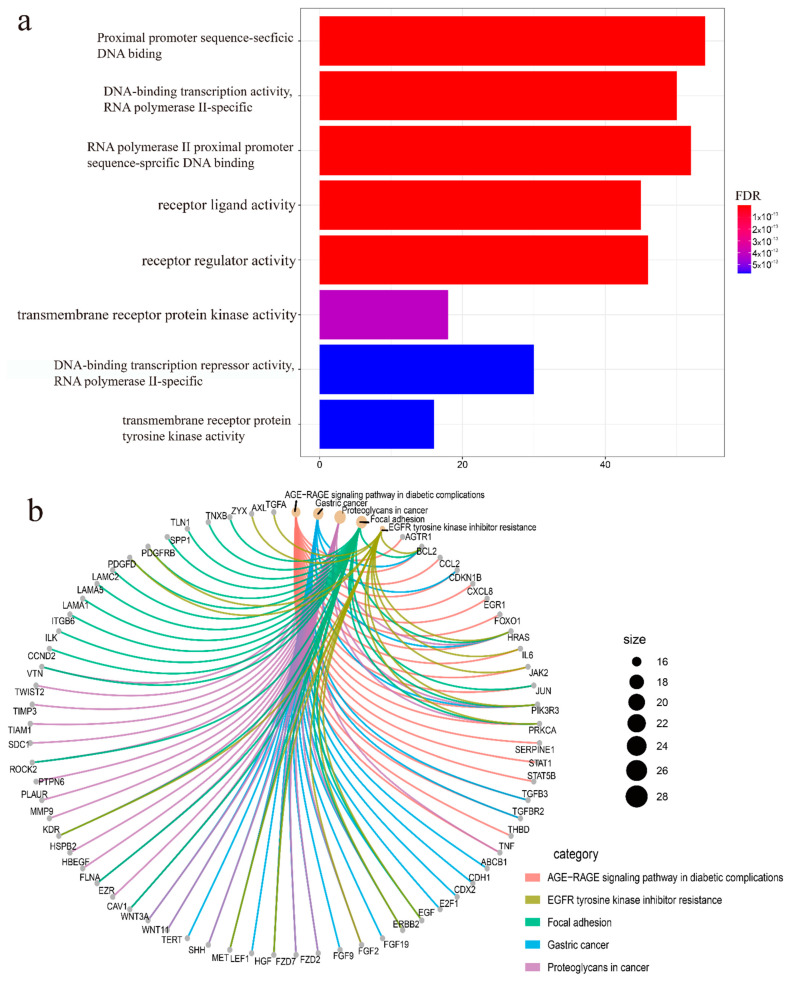
Enrichment analysis of differently expressed ERGs. (**a**) Gene Ontology pathway enrichment analysis of biological process among ERGs with a cut-off value *p* < 0.05, *p* was adjusted by the method of false discovery rate. (**b**) KEGG analysis among ERGs with a cut-off value *p <* 0.05, *p* was adjusted by the method of false discovery rate.

Biological functions pathways analysis was executed of these 405 differentially expressed ERGs. Gene ontology (GO) enrichment terms are shown in Figure 3a. The result of GO enrichment shows that the screened ERGs positively regulate the DNA-binding transcription activation in UCEC. KEGG pathway enrichment of these genes is shown in Figure 3b. These results indicate that the differentially expressed ERGs were mainly related to focal adhesion, EGFR tyrosine kinase inhibitor pathways, and transcriptional deregulation in cancer (Figure 3).

### 3.3. Screened for the ERGs with Significant Prognosis

The differentially expressed EMT-related genes (ERGs) in mRNA expression data of the UCEC cohort were identified by the “limma” package in R software (FDR < 0.05, |logFC| > 1). After combining clinical data and mRNA expression data of ERGs, univariate Cox regression analyses were used to screen the differentially expressed ERGs with remarkable prognostic value (*p <* 0.05). Additionally, 101 genes were screened with significant prognostic value in UCEC (Supplementary S1).

### 3.4. Establishment and Validation of the Prognostic EMT-Related Gene Signature

**Figure 4:**
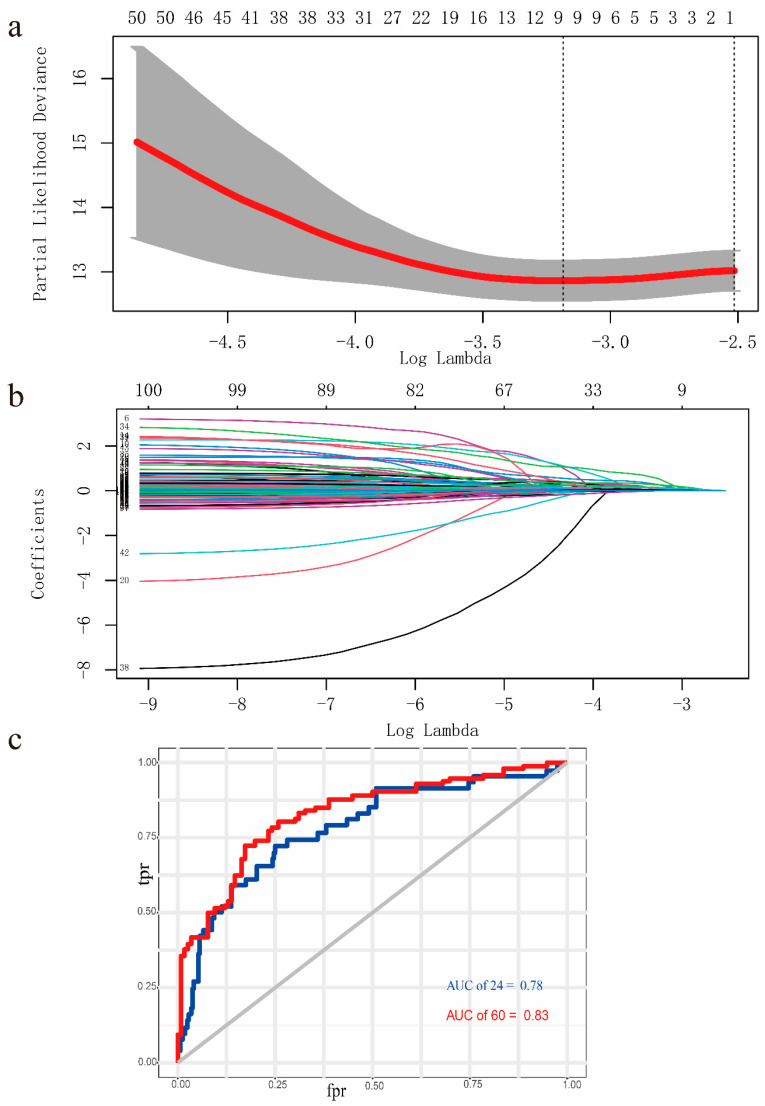
Construction of LASSO model. (**a**) Cross-validation for the penalty lambda. (**b**) LASSO regression coefficients over different values for penalty parameters. (**c**) AUC of the LASSO model constructed with the prognostic-related ERGs in the UCEC-TCAG cohort; AUC of the 2-year survival was 0.78, AUC of the 5-year survival was 0.83.

**Figure 5:**
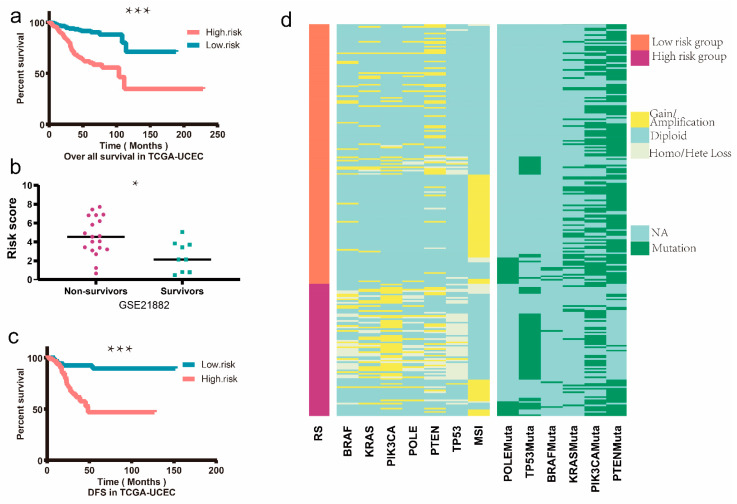
Survival analysis and heatmap. (**a**) In the TCGA-UCEC cohort, the 539 patients were divided into a high-risk group and a low-risk group according to the ERGs signature risk score median value, and the overall survival was compared between the two groups through Kaplan–Meier method, as cut-off *p* set as 0.05. (**b**) Patients in GSE21882 were divided into non-survivors and survivors after a 5-year follow-up. RS was statistically higher in the non-survivors group by the method of Wilcoxon rank sum test. (**c**) In the TCGA-UCEC cohort, the disease-free survival was compared in the high-risk group and low-risk group according to the gene signature risk score by the Kaplan–Meier method, as cut-off *p* set as 0.05. (**d**) Heat map of TCGA-UCEC patients, an overview of the copy number variation and mutation status of BRAF, KRAS, PIK3CA, PTEN TP53. MSI status was also displayed in the figure. * equals *p* < 0.05, *** equals *p* < 0.001.

To identify the EMT-associated genes, a machine learning algorithm, LASSO algorithm, was performed. We utilized 10,000-fold cross-validation to select penalty value, lambda (λ). In our study, the optimal computed lambda ranged from 0.0413–0.0809 (Figure 4a). We set the lambda at 0.0809 as it can generate a stricter penalty model which contains much fewer variates. At last, a 9-gene LASSO regression model was constructed (Figure 4b). The AUC of the constructed LASSO regression model was 0.83 when overall 5-year survival was applied and 0.78 when 2-year survival was applied. These 9 genes were selected for the next step analysis. Multivariate Cox regression analysis performed by the “survival” R package was subsequently used to construct a predictive signature. The final risk score was calculated as risk score = *EPHB2* ∗ 1.652937 + *TUFT1* ∗ 0.836239+ *CDKN2A* ∗ 0.062206 + *ONECUT2* ∗ 0.282455 + *RBP2* ∗ 0.129494 + *KLF8* ∗ 0.533818 + *E2F1* ∗ 0.158181 + *SIX1* ∗ 0.199476 + *ERBB2* ∗ 0.072221. The risk score of the prognostic signature was calculated and EC patients were divided into a high-risk group and a low-risk group according to median value. We verified this formula with overall survival data in both the TCGA-UCEC cohort and GEO dataset (GSE21882). Additionally, we found that this 9-gene signature was significantly associated with OS (Figure 5a). In GSE21882, as only survival status after 5 years could be acquired, we divided the patients into non-survivors and survivors, then the RS was compared between these two groups. Results showed that RS was significantly higher in the non-survivors group (Figure 5b). Additionally, risk score in TCGA-UCEC dataset were compared by Wilcoxon rank sum test in the 5-year survivors group and non-survivors group, result showed that the 5-year non-survivors group exhibited higher risk score, with *p* < 0.001 (Supplementary S2). In the TCGA-UCEC dataset, our analysis results showed that there was a significant statistical difference in the DFS between the low-risk and high-risk groups (Figure 5c).

Recently, the endometrial cancer molecular classification was introduced by TCGA and promoted by NCCN which initiated a transition toward molecular-based classification with significant prognostic value and a potential impact on the treatment of EC. There are four molecular subgroups: p53-abnormal (p53abn), *POLE*-ultra-mutated (*POLE*mut), mismatch repair-deficient (MMRd), and no specific molecular profile (NSMP). In our study, we explored the above molecular profiling of the high- and low-risk groups. In our study, there were higher copy number variations of *BRAF*, *KRAS*, *PIK3CA*, *PTEN*, and *TP53* in the high-risk group EC patients by the means of the chi-square test. Additionally, mutations rate of *P53*, *PTEN*, and *KRAS* were higher in the high-risk group of EC patients (Figure 5d). In the meantime, we analyzed the mutation rate and the CNVs of the 9 genes in our risk model, result showed that higher CNVs were found in the high-risk group of EC patients identified by our gene signature (Supplementary S3). Coordinated to the TCGA pathology and molecular analysis, high-risk group EC patients had a higher *P53* mutation rate and poorer prognosis.

**Figure 6:**
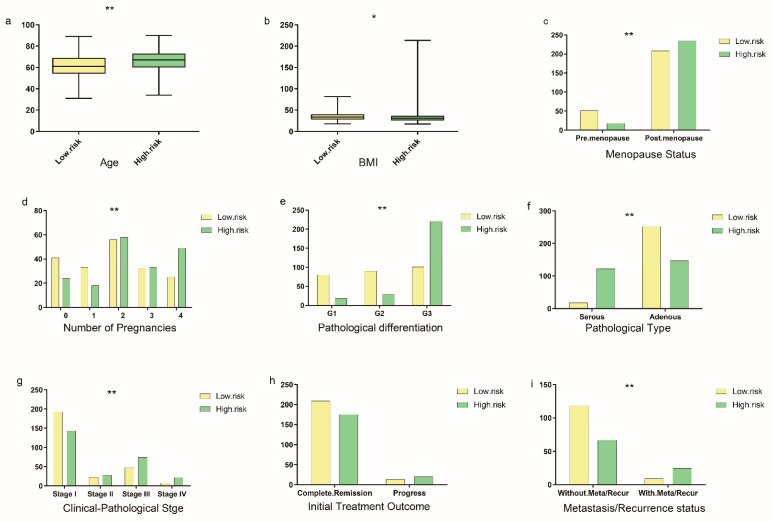
Risk score and clinical parameters. According to the calculated risk scores, the patients in the TCGA-UCEC cohort were divided into low-risk groups and high-risk groups. Additionally, different clinical characteristics were compared between the two groups with the chi-square test, including: (**a**) patients age at the time of diagnosis (*p* < 0.001); (**b**) patients BMI (*p* = 0.037); (**c**) different menopause status, pre-menopause status, and menopause status (*p* < 0.001); (**d**) number of pregnancies, 0–4 (*p* = 0.002); (**e**) different pathological differentiation (*p* < 0.001); (**f**) different pathological type (*p* < 0.001); (**g**) different clinical–pathological staging (*p* < 0.001); (**h**) different outcome after the initial treatment, complete remission or not (*p* = 0.105); (**i**) having recurrence/metastasis or not (*p* < 0.001): * equals *p* < 0.05; ** equals *p* < 0.01.

**Figure 7:**
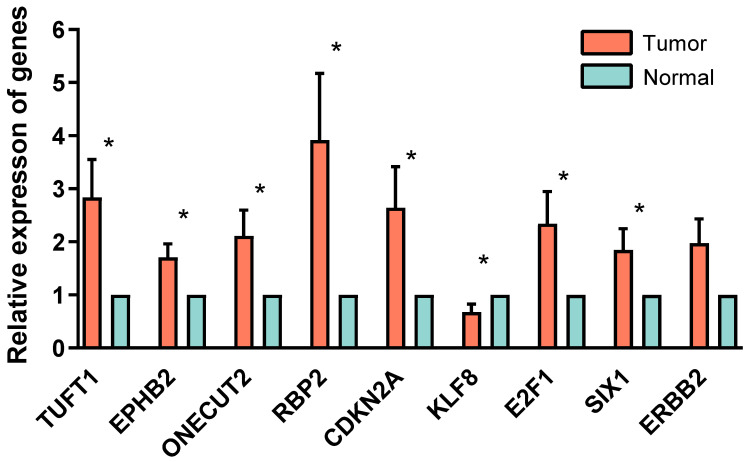
Quantitative real-time PCR. Relative expression of TUFT1, EPHB2, ONECUT2, RBP2, CDKN2A, KLF8, E2F1, SIX1, and ERBB2 in 10 pair-matched samples from endometrial carcinoma tissues versus normal endometrium tissues was compared by GAPDH RT-qPCR mean, SEM error bars. *t*-test was used to compared the relative expressions in normal and endometrial carcinoma tissue. * Equals *p <* 0.05.

The clinical–pathological characteristics were compared between the two groups, including patients’ age, BMI, pre-menopause/menopause status, number of pregnancies, different pathological grades, clinical–pathological staging, clinical outcome after the initial treatment, and metastasis/recurrence status. The cut-off *p* was set as 0.05. Additionally, the result showed that patients in the high-risk group had a higher percentage in menopause status, pregnancy no less than two times, G3 pathological differentiation, pathological type, advanced clinical–pathological staging, and recurrence or metastasis status (Figure 6). Besides, the expression of the ERGs in the risk model was validated by quantitative real-time PCR in clinical tissue specimens. All of the genes in the model were upregulated in endometrial carcinoma tumor tissue versus normal tissue except KLF8, which was consistent with the result derived from the TCGA database. Only the relative expression of *ERBB2* showed no statistically difference (*p* = 0.05), other genes expression all showed statistically difference between the tumor tissue and the normal tissue (*p* < 0.05) (Figure 7).

### 3.5. Nomogram Construction and Validation

**Figure 8:**
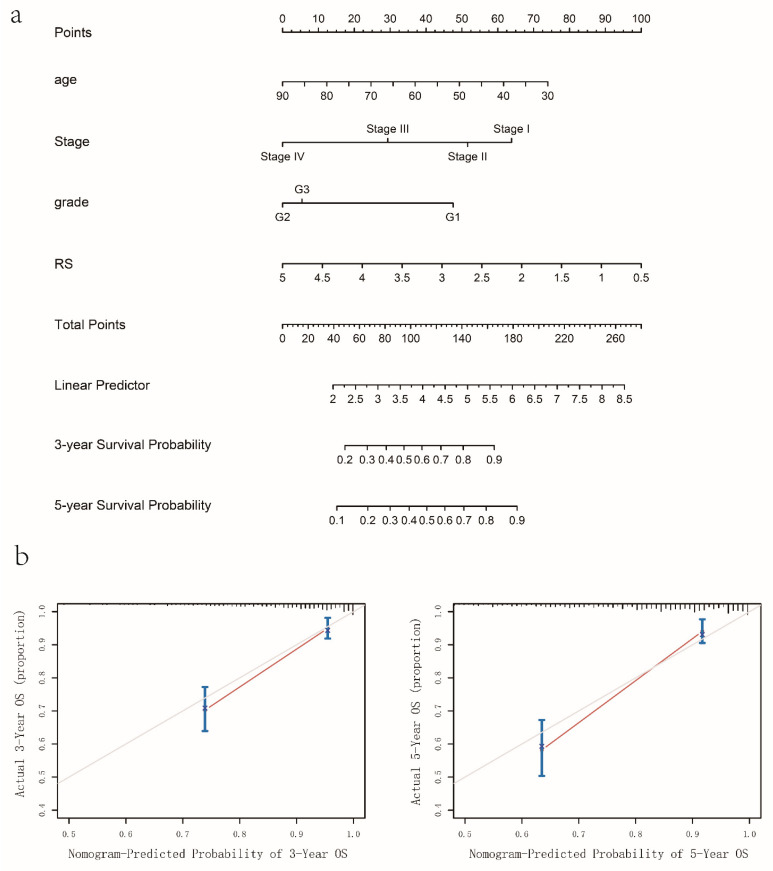
Nomogram and Calibration plot. (**a**) Nomogram constructed for the 3-year and 5-year predictive probability for the EC patients. (**b**) Calibration curve for the 3-year and 5-year predictive survival probability and actual survival proportion.

After univariate and multivariate survival Cox analysis, 4 significant prognostic parameters were enrolled in the construction of nomogram (*p* < 0.1 in multivariate cox regression), the detailed result was shown in Table 2. Respective points of age at diagnosis, differentiation grade, FIGO stage, and risk score can be acquired through the point scale axis (Figure 8a). The calculated C-index was 0.796 (95% CI: 0.700–0.893). Calibration curves were presented in Figure 8b, which showed that the predictive probability was in good accordance with the actual survival proportion.

### 3.6. Immune Microenvironment Analysis

**Figure 9:**
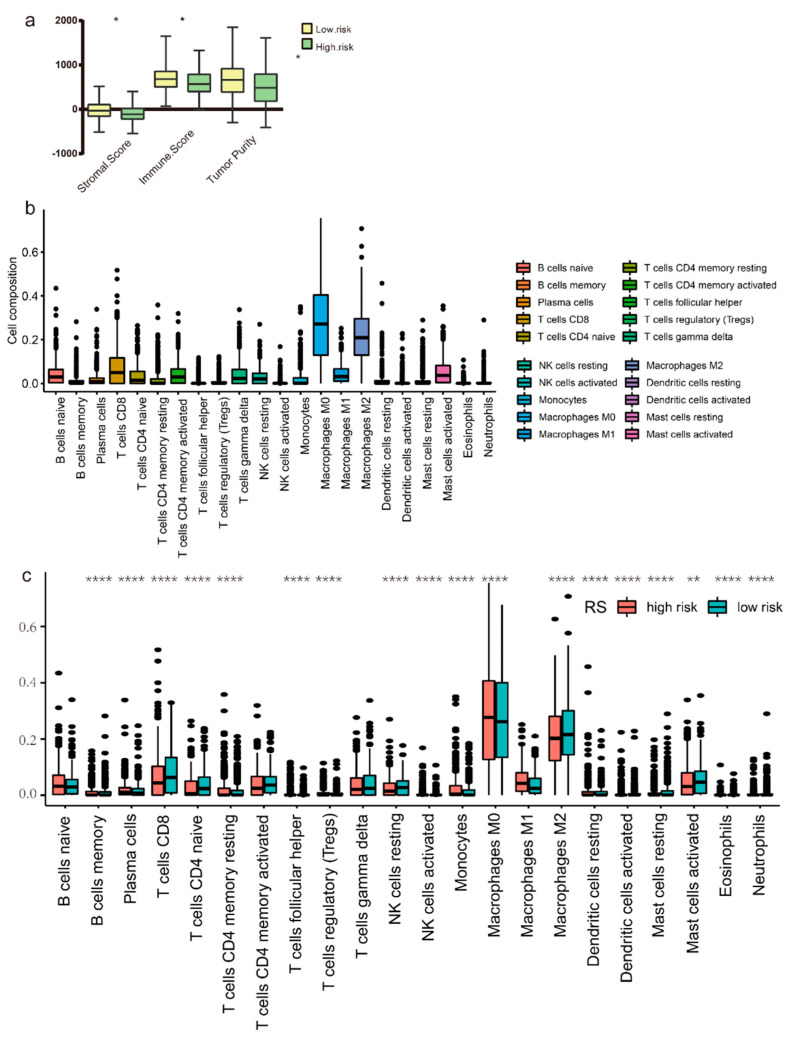
Immune microenvironment analysis. (**a**) High–risk score groups were associated with lower stromal scores, immune score, and tumor purity. Wilcoxon rank sum test was applied. * equals *p* < 0.05; ** equals *p* < 0.01; **** equals *p* < 0.001. (**b**) immune cell infiltration landscape in TCGA-UCEC patients. (**c**) Difference in immune cell infiltration abundance between high– and low–risk groups.

Based on the immune and stromal scores which were acquired through ESTIMATE, we analyzed their relation with the prognosis data in the TCGA-UCEC cohort. Wilcoxon rank sum test was used to compare the immune score, stromal score, and tumor purity between the high-risk and low-risk groups in the TCGA-UCEC cohort. All *p* values were lower than 0.05, demonstrating a significant difference in immune score, stromal score, and tumor purity between the groups (Figure 9a). The proportions of 22 tumor cells–immune cells were displayed in Figure 9b, and macrophage M0 and macrophage M2 were the top 2 components in EC tumor immune microenvironment. There was a significant difference between the high-risk and low-risk groups regarding to the proportion of the immune cells, and the result was shown in Figure 9c. Additionally, a higher proportion of macrophage M1 and lower macrophage M2 was found in the high-risk group, which indicate a potential benefit from the immune checkpoint blockades therapy.

### 3.7. Immune Checkpoint Blockades Therapy and Drug Sensitivity Prediction

**Figure 10:**
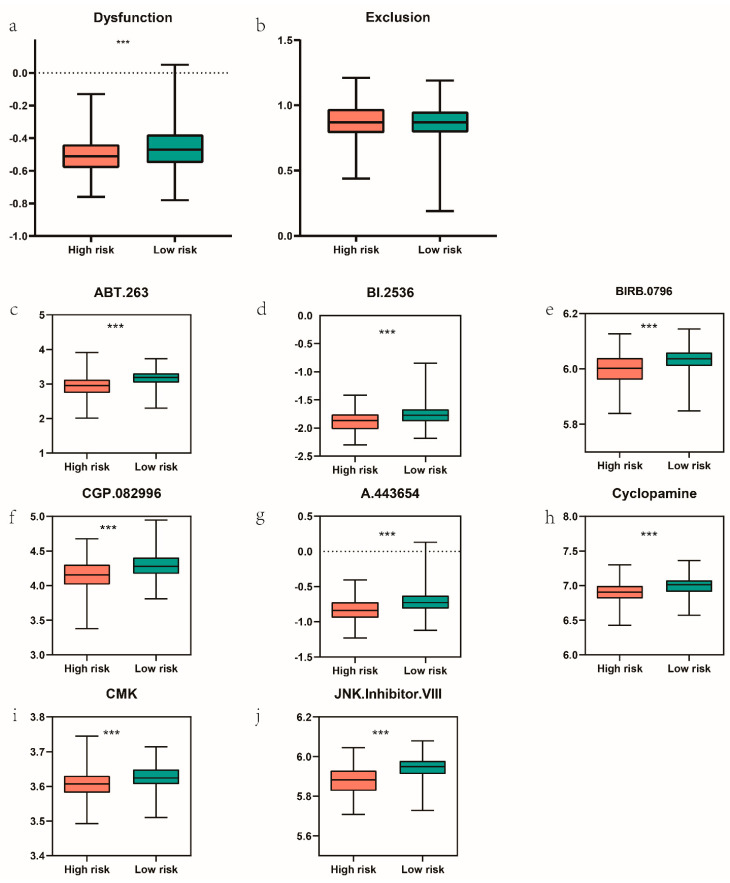
Immunotherapy and drug sensitivity prediction. (**a**,**b**) T cell dysfunction and T cell exclusion in the high– and low–risk patients with TCGA-UCEC. (**c**–**j**) Wilcoxon rank sum test was applied for comparing the different chemotherapeutic response predictions in high– and low–risk patients. *** equals *p* < 0.001.

Nowadays, immune checkpoint blockades targeting CTLA-4 and PD-1 has emerged as a promising strategy in various malignant tumor [21]. Clinical response to immune checkpoint blockades was estimated, and results show that T cells dysfunction was lower in the high-risk group, which indicate a more promising response to immune checkpoint blockades targeting CTLA-4 and PD-1 (Figure 10a), To obtain a better comprehensive analysis of chemotherapy response, drug sensitivity data were acquired from the GDSC database and the IC50 were compared. Pearson correlation between the risk score and IC50 of different chemical compounds was conducted, and 8 chemical compounds were identified for a negative correlation with the risk score (r < −0.3, *p* < 0.05). Results were displayed in Figure 10b. Among the 8 compounds, A.443654 is a selective Akt inhibitor. ABT.263 and BI 2536 both can induce apoptosis. Lower IC50 of these drugs indicates that high-risk EC patients were more sensitive to the treatment compared with the low-risk EC patients.

## 4. Discussion

It has been widely proved that EMT is an important biological process in cancer, including endometrial cancer. Specifically, EMT is characterized by downregulation of the epithelial cell properties and activation of the mesenchymal characteristic. EMT is thought to be one of the major mechanisms that determine the invasion and metastasis of cancer cells.

In this study, we collected the high-throughput data about the transcriptional expression of 543 EC samples and 35 nontumor samples with their corresponding clinical data from the TCGA database. At the same time, 1184 EMT-related genes (ERGs) were acquired from the Human EMT Database. Among these, we screened out the differentially expressed ERGs between EC samples and nontumor endometrium samples. Then, the differentially expressed ERGs were listed and analyzed, biological functions pathways analysis indicated that differentially expressed ERGs can promote cancer-related pathways, including focal adhesion and EGFR tyrosine kinase inhibitor pathways, and may lead to transcriptional misregulation in cancer. The AGE–RAGE axis is involved in the onset and progression of various chronic disorders and metabolic syndrome. Several recent researchers revealed an association of the AGE–RAGE signaling with different pathological conditions involved in the progression of cancer including cell proliferation, invasion, metastasis, and angiogenesis [24]. AGEs biomarkers pentosidine malondialdehyde (MDA), a lipoxidation product closely linked to AGEs, is suggested to be found in high amounts in breast cancer and lung cancer patients [25]. Our findings also imply that the AGE−RAGE signaling pathway may play a role in tumorigenesis of EC for the first time.

A novel machine learning method LASSO was used to finally identify 9 EMT-related genes, including *EPHB2*, *TUFT1*, *CDKN2A*, *ONECUT2*, *RBP2*, *KLF8*, *E2F1*, *SIX1*, and *ERBB2* as an independent prognostic predictor, and a formula about risk score (RS) was raised through multivariate Cox regression analysis. Some of these genes, such as *CDKN2A, E2F1*, and *ERBB2*, have been characterized in endometrial cancer tumorigenesis. *CDKN2A* is one of the crucial defenses against cancer development in several human cancers. *CDKN2A* was found to be inactivated in EC and its hypermethylation was correlated with an increased risk of EC [26,27]. The *HER2 (ERBB2)* gene is amplified in 17–33% of carcinosarcoma, uterine serous carcinoma, and a subset of high-grade endometrioid endometrial tumors [28,29]. However, for the remaining genes, their functions have not been revealed in EC. *TUFT1* was initially found to play an important role in the development and mineralization of tooth enamel and was later discovered in many cancerous tissues, including pancreatic cancer and hepatocellular carcinoma. Its expression was correlated with unfavorable clinicopathologic characteristics and poor survival [2]. Recent studies have shown that *TUFT1* promotes proliferation, metastasis, and epithelial–mesenchymal transformation of cancer cells through the Ca2+/PI3K/AKT pathway [30]. *ONECUT2* is a member of the one cut family of transcription factors, known to be an important regulator of early retinal cell fate during development. *ONECUT2* may play a critical role in pan-cancer-wide tumorigenesis [30]. RBP2 histone demethylase suppresses NOTCH signaling to sustain neuroendocrine differentiation and promote small cell lung cancer tumorigenesis [31]. Krüppel-like factor 8 (*KLF8*) is a critical inducer of EMT and invasion. *KLF8* induces EMT primarily by repressing E-cadherin transcription. *KLF8* promotes human breast cancer cell invasion and metastasis by transcriptional activation of MMP9 [32]. Sine oculis-related homeobox 1 homolog (*SIX1*) is a transcription factor that regulates the development of many tissues and becomes reactivated or overexpressed in multiple types of human cancer. Recent research showed that *SIX1* was essential for normal endometrial epithelial differentiation, and delayed DES-induced endometrial carcinogenesis by promoting basal differentiation of CK14þ/18þ cells [32]. Another study also indicated that the *SIX1* oncoprotein correlated with uterine cancer. *SIX1* was not present in normal endometrium but was expressed in a subset of endometrial cancers in patients who were also more likely to have late-stage diseases [33]. Further studies of the function of these genes may identify new drivers and therapeutic targets for endometrial cancer.

A prognostic EMT-related gene signature for EC specimens was constructed and EC patients were divided into high-risk and low-risk groups according to the risk score in each sample. The results showed that the risk score could successfully predict the survival of EC patients, in which the high-risk group had a significantly worse OS than the low-risk group. The ROC curves also indicated that the EMT-related gene signature had favorable prognostic power. All of these findings were validated in GEO data sets. In addition, we comprehensively analyzed the relationship of the EMT-related genes signature with different clinicopathological features. We found that our prognostic signature risk score was significantly correlated with clinicopathological data, including age, tumor grade, and tumor stage. We also constructed the nomogram by the age, grade, stage, and EMT signature that showed a good performance in predicting the survival of EC patients.

Tumors could acquire the possibility of aggressiveness and metastases through the EMT process [34]. Immune cells also participate in EMT via various pathways and in turn, cancer cells crosstalk with immune cells to release immunosuppressive substances to create an immunosuppressive microenvironment that promotes invasion and metastasis [35,36]. Then, we explored the immune microenvironments of the high- and low-risk group, and the high-risk group get a lower stromal score, immune score, and tumor purity. Immune infiltration analysis showed that macrophage M2 is lower and macrophage M1 is higher in cell fractions in the high-risk group, which indicated a proper microenvironment that enhanced T cells cytotoxic function.

Researchers had studied the whole gene expression signature that may indicated poor survival among the EC patients [37,38], and more evidence show that alterations in cancer genomes can strongly influence clinical response to anticancer therapies [23]. There were few studies reporting immunotherapy and EMT targeted therapy in EC patients, and certain EC patients can benefit from Pembrolizumab [39]. We further evaluated the likelihood of response to immunotherapy in the high- and low-risk groups. T cells dysfunction score by TIDE algorithm also indicated a promising response to immune checkpoint blockades therapy targeting CTLA-4/PD-1. As second-line chemotherapy was still debatable in advanced EC patients, we screened out eight compounds that may be crucial in the substitute chemotherapy in the GDSC database. A.443654 is a selective Akt inhibitor. PI3K/Akt/mTOR pathway is crucial in various tumors, including endometrium carcinoma. PTEN can regulate the tumor cells’ proliferation via the mTOR pathway, and 30–60% EC exhibits a lack of *PTEN*. Reports showed that mTOR inhibitors can improve disease-free survival in advanced EC patients [40]. ABT.263 disrupts Bcl-2/Bcl-xl interactions with pro-death protein, leading to the initiation of apoptosis, BI 2536 is a Plk1 inhibitor, which can induce apoptosis and attenuates autophagy. and BI 2536 both can induce apoptosis. Lower IC50 of these drugs indicates that high-risk EC patients may be more sensitive to these chemo treatments.

## 5. Conclusions

In conclusion, we identified and validated an EMT-related, 9-gene signature with certain prognostic values for EC patients. Immune microenvironment characters of the high- and low-risk groups were revealed, which may contribute to a better understanding of the molecular mechanism of EC and provide references for decision making during EC patients’ treatment.

## Figures and Tables

**Table 1:** Sequences of the primers.

Primer Name	Primer Sequence
*EPHB2* Foward	AGAAACGCTAATGGACTCCACT
*EPHB2* Reverse	GTGCGGATCGTGTTCATGTT
*TUFT1* Foward	TCAGACTGTGTGGCTTTTGAG
*TUFT1* Reverse	GTCAGCATTGTTGCTCCGAAG
*CDKN2A* Foward	GATCCAGGTGGGTAGAAGGTC
*CDKN2A* Reverse	CCCCTGCAAACTTCGTCCT
*ONECUT2* Foward	GGAATCCAAAACCGTGGAGTAA
*ONECUT2* Reverse	CTCTTTGCGTTTGCACGCTG
*RBP2* Foward	TTTTGCCACCCGCAAGATTG
*RBP2* Reverse	CGGAATGTGCTAGTGGTTTTTGT
*KLF8* Foward	CCCAAGTGGAACCAGTTGACC
*KLF8* Reverse	GACGTGGACACCACAAGGG
*E2F1* Forward	ACGCTATGAGACCTCACTGAA
*E2F1* Reverse	TCCTGGGTCAACCCCTCAAG
*SIX1* Forward	CTGCCGTCGTTTGGCTTTAC
*SIX1* Reverse	GCTCTCGTTCTTGTGCAGGT
*ERBB2* Foward	TGCAGGGAAACCTGGAACTC
*ERBB2* Reverse	ACAGGGGTGGTATTGTTCAGC
*GAPDH* Foward	AGATCCCTCCAAAATCAAGTGG
*GAPDH* Reverse	GGCAGAGATGATGACCCTTTT

**Table 2:** Age of diagnosis, FIGO stage, pathological grade, PLOE mutation status, P53 mutation status, MSI status, risk score, and immune score were analyzed by univariate Cox regression with the overall survival in TCGA-UCEC dataset. Then, those parameters with *p*-value lower than 0.5 were enrolled for multivariate Cox regression analysis.

Univariate Cox Regression				Multivariate Cox Regression		
Variates	*p*	HR	95% CI	*p*	HR	95% CI
Age	0.001	1.038	1.017–1.060	0.011	1.028	1.006–1.051
Stage	0.000	2.012	1.667–2.427	0.000	1.696	1.400–2.055
Grade	0.001	2.696	1.777–4.088	0.065	1.522	0.975–2.735
POLE mutation	0.293	0.463	0.111–1.941			
P53 mutation	0.329	1.411	0.707–2.817			
MSI	0.331	1.158	0.862–1.565			
Risk score	0.000	2.718	2.096–3.525	0.001	1.707	1.255–2.323
Immune score	0.023	1.001	1.000–1.002	0.166	1.001	1.000–1.001

## Data Availability

The datasets analyzed during the current study are available in The Cancer Genome Atlas (TCGA) and Gene Expression Omnibus (GEO). Epithelial–Mesenchymal Transition gene list was acquired from Epithelial–Mesenchymal Transition GENE database (http://dbemt.bioinfo-minzhao.org/ (accessed on 29 October 2020)). Tumor Immune Estimation Resource database (TIMER) was implied for associated immune analysis. Immune infiltration was acquired from CIBERSOT databases (https://cibersort.stanford.edu/ (accessed on 11 January 2021)). Tumor Immune Dysfunction and Exclusion (TIDE) and Genomics of Drug Sensitivity in Cancer (GDSC) were applied for immune checkpoint blockades therapy and drug sensitivity prediction separately.

## Supplementary Materials

The following supporting information can be downloaded at: https://www.mdpi.com/article/10.3390/genes13020216/s1, Supplementary S1: Univariate COX regression result; Supplementary S2: 5 years overall survival rate of TCGA-UCEC; Supplementary S3: Heat map of TCGA-UCEC patients, an overview of the copy number variation and mutation status of the 9 genes in the prognosis related gene signature.

## Abbreviations

EC	endometrial cancer
EMT	epithelial—mesenchymal transition
TCGA	The Cancer Genome Atlas
GEO	Gene Expression Omnibus
*EPHB2*	EPH receptor B2
*TUFT1*	tuftelin 1
*CDKN2A*	cyclin-dependent kinase inhibitor 2A
*ONECUT2*	one cut homeobox 2
*RBP2*	retinol-binding protein 2
*KLF8*	Kruppel-like factor 8
*E2F1*	E2F transcription factor 1
*SIX1*	SIX homeobox 1
*ERBB2*	Erb-b2 receptor tyrosine kinase 2
RS	risk score
ESTIMATE	estimation of stromal and immune cells in malignant tumor tissues using expression data
ICB	immune checkpoint blockade
TIDE	tumor immune dysfunction and exclusion
GDSC	Genomics of Drug Sensitivity in Cancer
ERGs	EMT-related genes
UCEC	uterine corpus endometrial carcinoma
ROC	receiver-operating characteristic
TME	tumor microenvironment
LASSO	least absolute shrinkage and selection operator
IC50	the half-maximal inhibitory concentration
FDR	false discovery rate
MMRd	mismatch repair-deficient
CTLA-4	cytotoxic T-lymphocyte antigen 4
PD-L1	programmed cell death-ligand 1
FIGO	The International Federation of Gynecology and Obstetrics
AUC	area under the curve
OS	overall survival
DFS	disease-free survival
