# Supplementary figures and images for: Identification of a Novel Epithelial–Mesenchymal Transition-Related Gene Signature for Endometrial Carcinoma Prognosis
Source: Genes (Basel). 2022 Jan 25;13(2):216. doi: 10.3390/genes13020216 (PMC8872195; doi:10.3390/genes13020216)

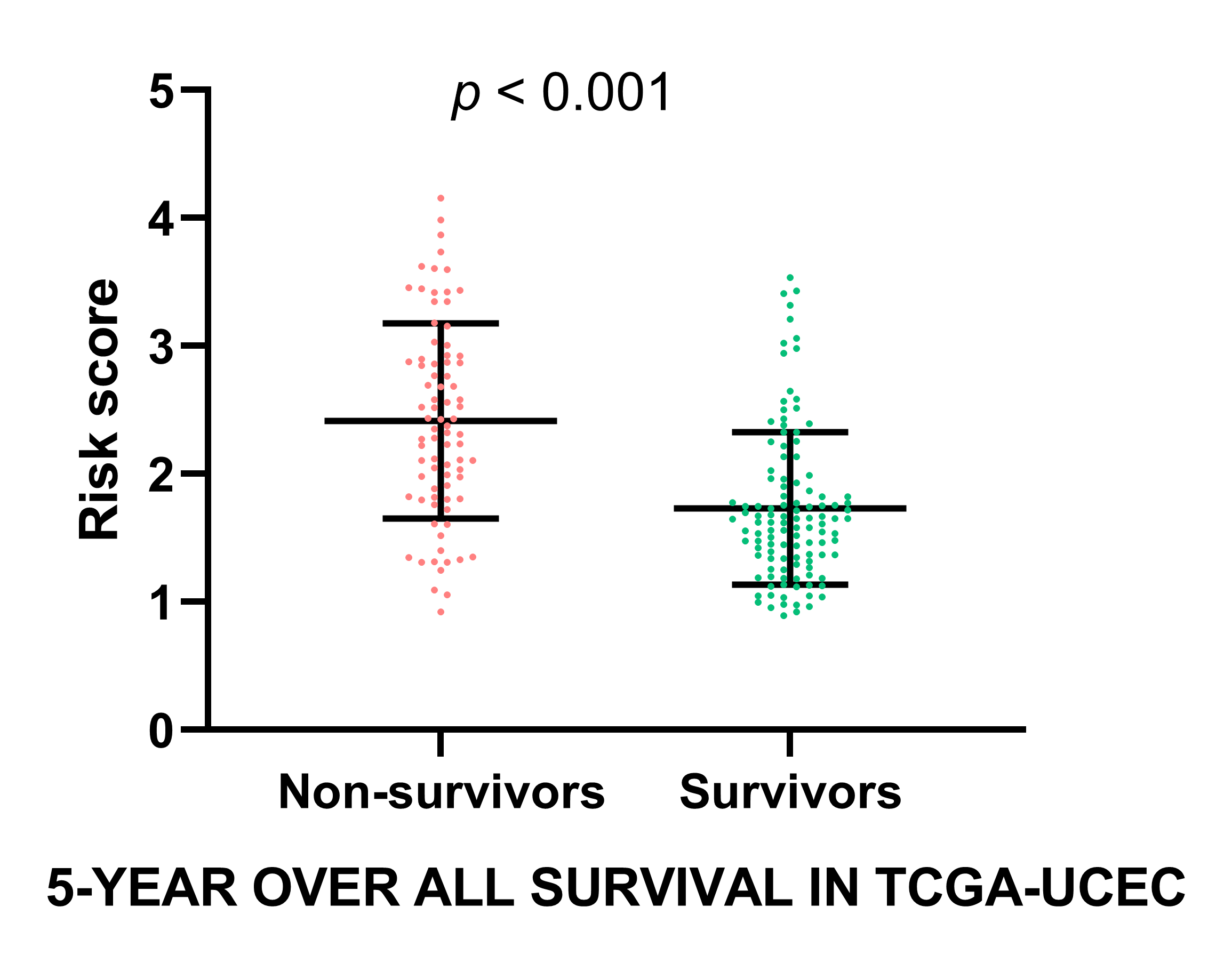

Supplement: Supplementary file 1 [file genes-13-00216-s001.zip › genes-1506677-supplementary/supplementary/Supplementary S2.tif]
